# Norwegian honey bees surviving *Varroa destructor* mite infestations by means of natural selection

**DOI:** 10.7717/peerj.3956

**Published:** 2017-10-24

**Authors:** Melissa A.Y. Oddie, Bjørn Dahle, Peter Neumann

**Affiliations:** 1 Vetsuisse Faculty/University of Bern, Institute of Bee Health, Bern, Switzerland; 2 Norwegian University of Life Sciences, Department of Animal and Aquacultural Sciences, Ås, Norway; 3 Agroscope, Swiss Bee Research Center, Bern, Switzerland

**Keywords:** *Apis mellifera*, Honey bees, *Varroa destructor*, Mites, Parasites, Natural selection

## Abstract

**Background:**

Managed, feral and wild populations of European honey bee subspecies, *Apis mellifera*, are currently facing severe colony losses globally. There is consensus that the ectoparasitic mite *Varroa destructor*, that switched hosts from the Eastern honey bee *Apis cerana* to the Western honey bee *A. mellifera*, is a key factor driving these losses. For >20 years, breeding efforts have not produced European honey bee colonies that can survive infestations without the need for mite control. However, at least three populations of European honey bees have developed this ability by means of natural selection and have been surviving for >10 years without mite treatments. Reduced mite reproductive success has been suggested as a key factor explaining this natural survival. Here, we report a managed *A. mellifera* population in Norway, that has been naturally surviving consistent *V. destructor* infestations for >17 years.

**Methods:**

Surviving colonies and local susceptible controls were evaluated for mite infestation levels, mite reproductive success and two potential mechanisms explaining colony survival: grooming of adult worker bees and Varroa Sensitive Hygiene (VSH): adult workers specifically detecting and removing mite-infested brood.

**Results:**

Mite infestation levels were significantly lower in surviving colonies and mite reproductive success was reduced by 30% when compared to the controls. No significant differences were found between surviving and control colonies for either grooming or VSH.

**Discussion:**

Our data confirm that reduced mite reproductive success seems to be a key factor for natural survival of infested *A. mellifera* colonies. However, neither grooming nor VSH seem to explain colony survival. Instead, other behaviors of the adult bees seem to be sufficient to hinder mite reproductive success, because brood for this experiment was taken from susceptible donor colonies only. To mitigate the global impact of *V. destructor*, we suggest learning more from nature, i.e., identifying the obviously efficient mechanisms favored by natural selection.

## Introduction

The European honey bee, *Apis mellifera*, is an economically important insect, providing essential pollination services for human food security as well as valuable hive products for the apicultural sector (Morse & Calderone, 2000; Klein et al., 2007). A honey bee colony is considered a superorganism and employs a series of social immunity strategies to optimize health and fitness; individuals within the colony perform hygienic behaviors to reduce risk of disease and parasite invasion (Seeley, 1989; Cremer, Armitage & Schmid-Hempel, 2007). However, major losses of managed and feral *A. mellifera* colonies have been well-documented in recent years (e.g., Kraus & Page, 1995; Neumann & Carreck, 2010; Van Engelsdorp et al., 2011; Pirk et al., 2014). The ectoparasitic mite, *Varroa destructor* (Anderson & Trueman, 2000) (originally infesting the Eastern honey bee *Apis cerana*) now infests *A. mellifera* near globally (Ellis & Munn, 2005). There is consensus that this mite is the main biotic factor threatening *A. mellifera* colony survival (Neumann & Carreck, 2010; Rosenkranz, Aumeier & Ziegelmann, 2010). The mite is a very efficient vector of several honeybee viruses, generating a disease epidemic within the colony. This, coupled with the exponential growth of mite populations sustained by developing host worker brood throughout the year and additional seasonal male brood (Rosenkranz, Aumeier & Ziegelmann, 2010; Dietemann et al., 2012) will cause a colony to dwindle until it dies in 2–3 years (Neumann et al., 2012).

Despite these drastic effects of *V. destructor* on *A. mellifera* host populations, there are reports of managed and feral *A. mellifera* honey bee populations that have survived mite infestations by means of natural selection. These populations have now been documented for more than 10 years (Avignon and Le Mans, France, Le Conte et al., 2007; Island of Gotland, Sweden, Fries, Imdorf & Rosenkranz, 2006; Arnot Forest, USA, Seeley, 2007; reviewed by Locke, 2016). In Gotland and Avignon, reduced mite reproductive success has been observed (Locke, 2016), which can contribute to colony survival. Up until now however, the mechanisms enabling the survival of mite-infested colonies have not been identified.

Two behavioral mechanisms of social immunity have been suggested to contribute to *V. destructor* survivability: one targets the mites at the phoretic stage, where it feeds on adult host bees, and one targets the reproductive stage, when the mites are sealed in cells with host brood. The former occurs when adult worker bees remove phoretic mites from themselves and/or nestmates via autogrooming and allogrooming (Guzman-Novoa et al., 2012). The latter describes adult worker bees detecting and removing mite-infested brood and has been defined as part of Varroa Sensitive Hygiene (VSH). The removal of infested brood inhibits contribution of these mites to the next generation and reduces the in-colony population (Harbo & Harris, 2009; Harris, Danka & Villa, 2010; Harris, Danka & Villa, 2012). Taken together, these two behaviors might explain reduced *V. destructor* reproductive success and ultimately explain colony survival. However, data from natural surviving populations remain scarce.

It is known that a managed population of local honey bees has been surviving for >17 years with no mite treatment in the Østlandet region of Norway. Mite levels were anecdotally low, despite the population being within sufficient distance of known susceptible colonies from various backgrounds (mostly *A. m. mellifera*, *A. m. carnica*, Buckfast) that would facilitate horizontal parasite transfer. The aim of this study was to estimate mite infestation levels and mite reproductive success in this surviving population with comparisons to a local and known-susceptible population. It also investigated the two aforementioned mechanisms for colony survival by quantifying grooming and brood removal (VSH) in both surviving and susceptible colonies.

## Materials & Methods

Experiments were conducted in the Østlandet region, Norway, during local late summer and early fall 2015. Surviving colonies were of a mixed origin (Buckfast) that had been kept without any *V. destructor* treatments for 19 years prior to the study. After the last treatment against *V. destructor* in 1997, mite levels seemed to increase and substantial losses of colonies occurred. However, surviving and healthy colonies were split and used to replace lost ones. Over the last 10 years, colony losses have been lower than the national average of about 10%. Susceptible local control colonies were located ∼60 km away from the surviving apiaries in a local *A. m. carnica* conservation area and treated against *V. destructor* on a biannual basis. We did not collect any genetic data to verify the actual racial admixture rates of either surviving or susceptible colonies used in the experiments.

### Mite infestation levels and proportions of damaged mites (grooming)

Daily mite drops were considered a viable measure of population size (Flores, Gil & Padilla, 2015) as none of the experimental colonies in the year of study had been given treatment against *V. destructor*. Rates were estimated using standard methods (Dietemann et al., 2013): The bottoms of the colonies were equipped with a mesh divider separating the mite board from the brood box and boards were prepared with paper towel soaked in vegetable oil to prevent scavenging of fallen mite bodies by ants (Dainat et al., 2011). The boards were placed under the test colonies and collected again six days later. Once the boards were collected, all mites were counted. The total mite numbers were then divided by the number of days the boards were left out and averaged across the colonies to give the mean daily drop rate for both surviving and susceptible colonies.

The proportion of damaged mites was used to estimate levels of grooming within a colony. Up to 20 mites from each colony were examined under a dissecting microscope and damage to the carapace, ventral plate and legs was noted in line with methods used by Rosenkranz et al. (1997). Each mite received a binary score of ‘damaged’ or ‘undamaged’ for the analysis and a proportion of damaged mites was obtained for each apiary.

### Varroa-sensitive hygiene (VSH)

One surviving and one control apiary were selected: five colonies from each received two brood frames from one of ten susceptible, local, donor colonies in a separate apiary, which was geographically distinct from that of the surviving and control test apiaries (∼60 km) and similarly untreated that year. Initial mite infestation levels in all test colonies were recorded two months prior. Only worker brood was considered for this study as male sexual (drone) brood is generally scarce during mite population peaks.

The ten susceptible donor colonies were chosen for their evidence of high mite loads. Each of these external source colonies donated one worker brood frame to a surviving and a susceptible receiver colony (*N* = 2 in total). Prior to frame relocation, the queens of these colonies were caged on each of the two empty frames for a period of two days to obtain defined age cohorts of brood. Frames were removed from the source colonies as soon as the brood was capped. Brood patches were then photographed and mapped on both sides to record brood patterns before being transferred to the receiver colonies. Frames were placed into the center of the brood chamber and left in the colonies for a period of 10 days to allow for a maturation point of ∼24 h prior to adult emergence (Winston, 1991). After the allotted time, frames were removed and photographed again before being transferred to storage at −20 °C before examination.

Each cell opened was mapped on the printed photograph of the brood comb and marked ‘infested’ or ‘uninfested’. If a cell had been cleaned and left empty by the bees this was also marked, determined by comparing the new photographs to those taken before frames were inserted into the test colonies. The number of empty cells was taken as a proportion of the total number of cells examined on the frame. This measure together with the mite infestation rates (Harris, 2007) were used to assess the level of VSH in surviving and susceptible colonies.

### Mite reproductive parameters

A subset of cells on these frames were examined in more detail to obtain levels of mite reproductive success. Once a cell was opened, the bee pupae were removed using fine forceps. Mites clinging to the body were brushed off with a small paint brush. The cell interior was also brushed carefully to extract, but not damage the remaining mites and eggs. Once all contents had been removed from the cell, the developmental stage of each mite was noted according to Martin (1994).

The measure of mite reproductive success was evaluated as the potential number of viable female offspring produced per foundress mite. Offspring were only considered viable if they were of an adequate stage to survive upon host emergence and if at least one male was present within the cell (Corrêa-Marques et al., 2003; Locke et al., 2012).

All cells that did not have daughter mites meeting these requirements were given a value of zero. For every colony, the average number of viable female offspring per foundress was found by counting the female offspring produced in one cell and dividing it by the number of foundresses in that cell. The brood stage was estimated based on a visual chart by Martin (1994) and pupae were assigned a number from 7 to 12 loosely based on the number of days each stage is commonly associated with. Brood younger than stage 7 (>170 h capped) was not considered.

### Statistics

R statistical analysis software (R Core Team, 2014) and the LME4 package (Bates et al., 2015) were used to perform statistical analyses. The daily mite drop of colonies was averaged for surviving and susceptible groups and comparisons were made using a two-sample *t*-test. To accommodate large outliers, data was log-transformed before statistical analyses were carried out.

Proportions of damaged mites were collected and the total proportion of infested cells as well as the proportion of cells hygienically removed by the bees were pooled by treatment and compared using 2 × 2 chi-squared contingency tests. A general linear mixed effects model (Bolker et al., 2009) was performed for mite reproductive parameters. Models were fitted by maximum likelihood and non-significant terms were removed progressively to acquire the minimum adequate model that best described the data. Parameters were averaged by frame. Donor colony ID as well as receiver colony type (surviving or susceptible) were accounted for as variables to include the paired design effect (a donor colony providing one frame to both treatment groups) as well as the receiver colony-level variation. The full model is expressed below:

\begin{eqnarray*}d1\lt -lmer(fecund\sim trt.col+avg.brood.stage+(1{|}origin.col)) \end{eqnarray*}

where ‘fecund’ is the average number of viable female offspring, ‘trt.col’ is the population type (Surviving or Susceptible), ‘avg.brood.stage’ is the average brood stage of the cells analyzed on that frame and ‘origin.col’ is the donor colony ID.

Mite reproductive success is known to decrease with a higher number of foundresses in a cell (Fuchs & Langenbach, 1989; Martin, 1995) and the potential offspring estimate error is larger in younger stages of brood (Locke et al., 2012). Both parameters were accounted for. Models were adjusted for the count response variable using a Poisson error structure. Dispersion was accounted for in GLMM using the package blmeco (Korner-Nievergelt et al., 2015).

## Results

One frame in the group of surviving colonies did not contain any brood after the 24-h queen-caging period and was therefore excluded. The distributions of the number of foundresses per cell were compared between surviving and susceptible colonies using a Kolmogorov–Smirnov test and found to be sufficiently similar that they did not need to be added to the models as a fixed effect (*D* = 0.08, *p* = 0.49).

**Figure 1:**
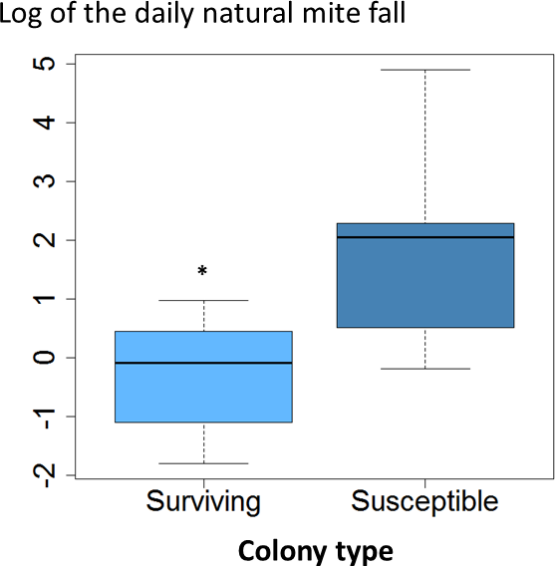
Daily natural mite fall in surviving and susceptible colonies. Interquartile ranges and medians of daily natural mite fall are shown. Values were log-transformed to accommodate outliers. Natural mite fall was significantly lower in surviving colonies compared to susceptible colonies (*t* = 3.8, *df* = 15, *p* < 0.002; *, *p* < 0.05).

**Figure 2:**
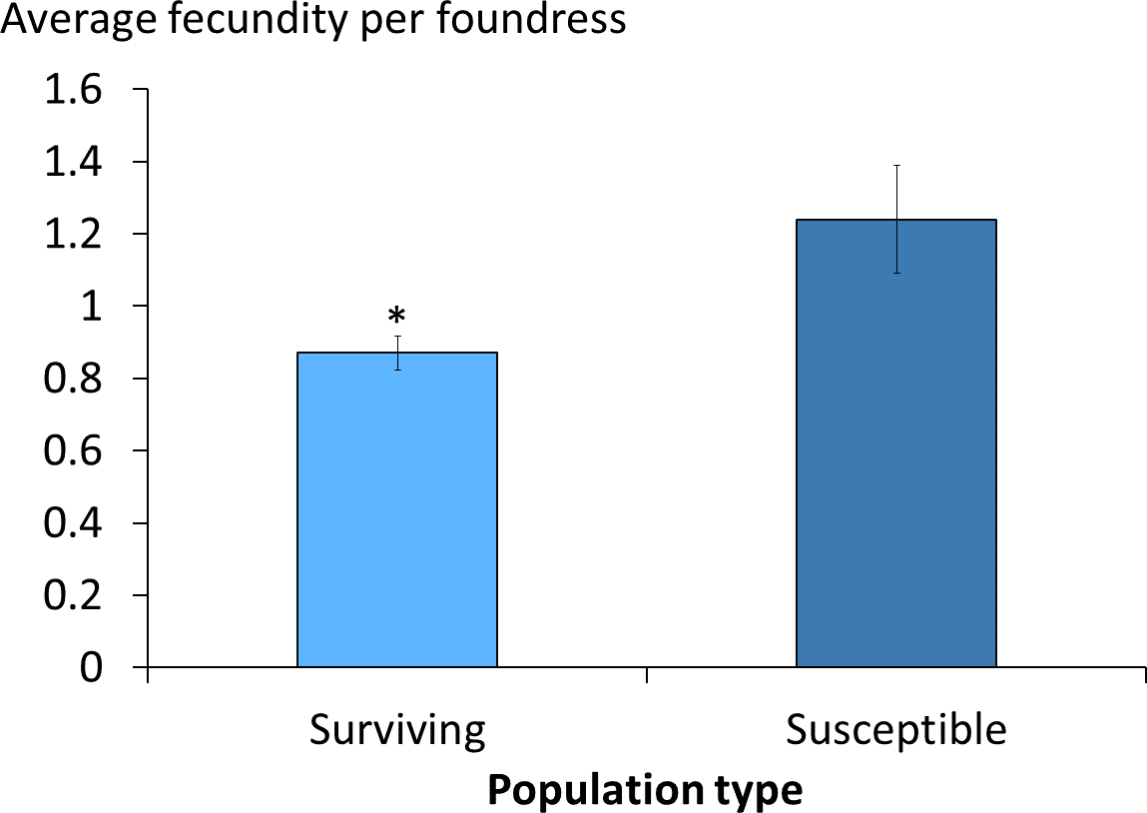
Viable female mite offspring per foundress in surviving and susceptible colonies. The average number and standard errors are shown. The frames in the surviving colonies had a significant decrease in mite reproductive success. Success was ∼30% lower when compared to susceptible colonies (*χ*^2^ = 4.09, *p* = 0.027; *, *p* < 0.05).

The average daily mite drop counts were significantly lower in surviving colonies compared to susceptible ones (Fig. 1A. *t* = 3.8, *df* = 15, *p* = 0.002). The overall average mite reproductive success in surviving colonies was significantly reduced at 0.87 offspring per foundress whereas in susceptible colonies it was 1.24. The reduction in mite reproductive success is ∼30% (Table 1, Fig. 2. *χ*^2^ = 4.09, *p* = 0.027).

**Figure 3:**
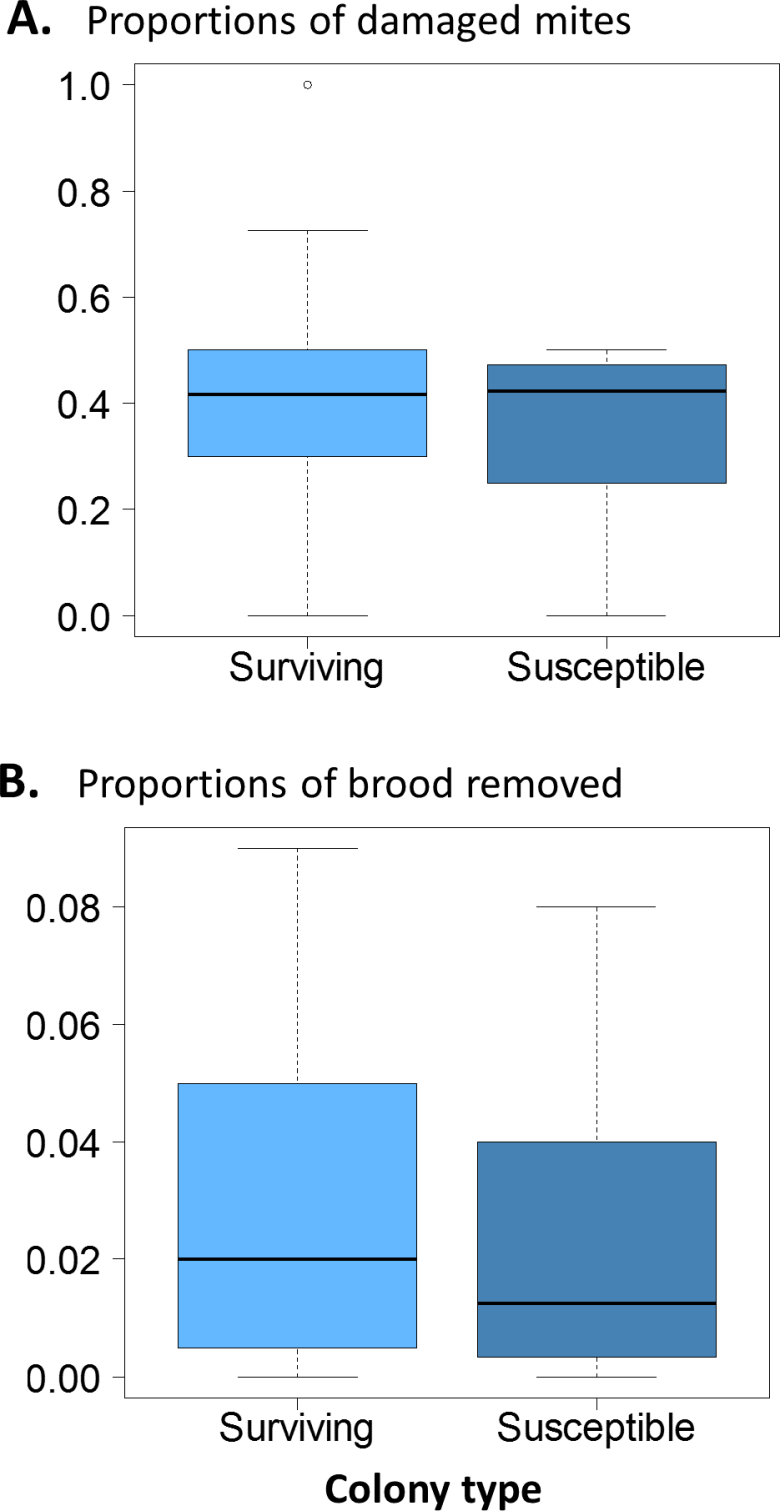
(A) Proportions of damaged mites in surviving and susceptible colonies. Interquartile ranges and medians are shown. There was no significant difference between surviving or susceptible colonies (*χ*^2^ = 0.12, *df* = 1, *p* = 0.73). (B) Proportions of brood removed in surviving and susceptible colonies over a period of 10 days. Interquartile ranges and medians are shown. There was no significant difference between surviving and susceptible apiaries (*χ*^2^ = 1.88, *df* = 1, *P* = 0.171).

There were no significant differences in the proportion of damaged mites between the surviving and susceptible colonies (Fig. 3A. *χ*^2^ = 0.12, *df* = 1, *p* = 0.73); ∼40% of the mites collected were damaged in both groups. Similarly, brood removal rates (VSH) were not significantly different between the surviving and susceptible colonies (Fig. 3B. *χ*^2^ = 1.88, *df* = 1, *P* = 0.171) with rates resting close to 5%. The proportion of infested cells, when compared between groups was slightly higher in surviving colonies (*χ*^2^ = 9.91, *df* = 1, *p* = 0.002).

**Table 1:** Output of the general linear mixed effects models used to analyse the average number of viable female offspring (fecundity), the brood infestation rate and the proportion of cells removed (VSH).

Response variable	Explanatory variable	*n*	*χ*^2^	*P* Value
Fecundity	Brood stage	19	0.38	0.54
	Colony type		4.90	0.027^*^

**Notes.**

* indicates significance.

## Discussion

Our data support the view that reduced *V. destructor* reproductive success is a prime requisite for natural survival of infested *A. mellifera* colonies. Indeed, both mite reproductive success and mite population levels were significantly lower in surviving Norwegian colonies compared to local susceptible controls. The proportions of damaged mites as a proxy for the efficacy of grooming behavior and brood removal (VSH) by adult workers were not significantly different between surviving and susceptible colonies, indicating that these two mechanisms are unlikely to explain the natural survival of these mite-infested Norwegian honey bee.

The mite population levels, as estimated by bottom board counts, were significantly lower in surviving colonies compared to local susceptible controls. This result is well in line with earlier findings for other surviving *A. mellifera* populations (Rosenkranz, 1999, reviewed by Locke, 2016). Lower mite infestation levels are an obvious explanation for colony survival and can result from reduced mite reproductive success. Indeed, only about half of the mites in Gotland colonies successfully produced viable mated daughter mites that contribute to the colony’s mite population, compared to ∼80% in local susceptible colonies (Locke & Fries, 2011). Similarly, mite reproductive success in the Avignon mite-surviving population was also reduced by 30% compared to local mite-susceptible colonies (Locke et al., 2012). Our data also show a mite reproductive success reduced by ∼30%, thereby strongly suggesting that such a reduction is sufficient to enable colony survival. It appears essential therefore to understand the mechanisms driving the reduced mite reproductive success.

Even though higher levels of grooming behavior have been shown to reduce *V. destructor* infestations in *A. mellifera* (Guzman-Novoa et al., 2012), our results show no significant differences for grooming or VSH between local surviving and susceptible Norwegian bees. This agrees well with earlier findings for Gotland, where differences in hygienic and grooming behavior were not apparent between the local surviving and mite-susceptible colonies (Locke & Fries, 2011). Reduced reproductive success in surviving Norwegian and Swedish colonies is not likely due to a more sensitive grooming threshold nor a higher level of brood removal (VSH). Neither of the tested traits seem to play a major role inlocal colony survival.

Since only susceptible donor brood was used for our experiments in both surviving and susceptible host colonies, any traits of immature bees can safely be excluded to explain our data. For example, changes in brood volatiles (Nazzi & Le Conte, 2016) are not a factor in the results obtained. Instead, it appears that different adult behaviours are likely sufficient to explain reduced *V. destructor* reproductive success and ultimately colony survival. These behaviors need to be identified.

When examining the total proportion of infested cells in the donor frames given to surviving and susceptible colonies it was found that frames for the surviving colonies had a higher number of infested cells (∼10%). We cannot in this study confidently attribute the difference in infestation rate to the differences between surviving or susceptible groups for several reasons: 1. This result does not align with our confirmed finding of low mite numbers in bottom board counts. 2. This result could be the fault of a low frame number and high variability in mite loads or the method of selecting frames from the donor colonies. In the future, differences in infestation rate between surviving and susceptible populations should be monitored with a larger sample size to explore the validity of this finding.

In conclusion, our data support the claim that a reduced *V. destructor* mite reproductive success seems to be a key factor in natural colony survival. However, grooming and VSH are unlikely for this Norwegian case. Instead, yet unidentified behavioral traits of worker bees seem sufficient to explain reduced mite reproductive success. The underlying mechanisms remain elusive and should be a focus of future studies taking advantage of naturally-selected survivors.

This Norwegian honey bee population, taken together with previously reported independent cases (Locke, 2016) clearly show that European honey bee subspecies can indeed develop traits to overcome extreme *V. destructor* infestations by means of natural selection. It is therefore high time we take advantage of these cases and gain a better understanding of natural host adaptations (Fries & Bommarco, 2007) for a practical application in apiculture and honey bee conservation worldwide.

##  Supplemental Information

10.7717/peerj.3956/supp-1 Data S1 Metadata describing column names and command scripts created for statistics in R Listed first are the descriptions for each column name in each dataset. Listed second are all scripts used and outputs drawn from the statistical program R.

10.7717/peerj.3956/supp-2 Data S2 Average fecundity, varroa-sensitive hygiene and infestation rates A dataset containing the fecundity measures for varroa mites, the level of brood removal (VSH) and the proportion of infested cells determined on all test frames.

10.7717/peerj.3956/supp-3 Data S3 Mite grooming proportions Dataset containing the proportion of damaged mites (grooming measure) found within all test colonies.

10.7717/peerj.3956/supp-4 Data S4 Mite bottom board counts A dataset containing varroa mite population estimates via bottom board counts.

10.7717/peerj.3956/supp-5 Data S5 Distribution of multiple foundress infestation events A dataset containing the distribution of the varying number of foundresses infesting single cells.

## Additional Information and Declarations

### Data Availability

The following information was supplied regarding data availability: The raw data sets have been supplied as Supplemental Files.
